# Clinical Presentation and Treatment Patterns of Pediatric Epistaxis: A Single-Center Study

**DOI:** 10.7759/cureus.54309

**Published:** 2024-02-16

**Authors:** Thamer Alshami Marghel Alruwaili, Yazeed Mayah Alazmi, Meshari Mosleh Alenzi, Noha Farouk Tashkandi

**Affiliations:** 1 Pediatrics, College of Medicine, Jouf University, Sakaka, SAU; 2 General Practice, College of Medicine, Jouf University, Sakaka, SAU; 3 Medical Research, College of Medicine, King Saud bin Abdulaziz University for Health Sciences, Riyadh, SAU

**Keywords:** saudi population, treatment patterns, clinical presentation, epistaxis, pediatric

## Abstract

Background: Epistaxis, commonly known as nose bleeding, is a prevalent condition in pediatric patients, often managed either at home or in clinical settings. This study aimed to explore the differences in the management of pediatric epistaxis between home and clinical settings, focusing on gender distribution, clinical presentations, and treatment methods.

Methods: A retrospective review was conducted, analyzing pediatric epistaxis cases managed both at home and in clinical settings. Data on gender distribution, clinical presentation, and treatment methods were collected and analyzed. Home remedies, first aid management, and clinical interventions like the use of nasal sprays and septoplasty were evaluated.

Results: The study found significant differences in gender distribution between home (46.2% males) and clinical settings (61% males). Recurrent nasal bleeding was more common in home settings (75%), whereas more complex cases were predominant in clinical settings. Nasal sprays containing decongestants were widely used in clinical settings (62.1%), contrasting with a preference for first aid measures at home. Surgical interventions like septoplasty were occasionally employed in clinical scenarios.

Conclusion: The study highlights distinct approaches to managing pediatric epistaxis in home versus clinical settings. It underscores the importance of tailored treatment strategies, considering the severity and frequency of epistaxis episodes. These findings suggest a need for comprehensive guidelines to assist caregivers and healthcare professionals in effective decision-making for pediatric epistaxis management. The study also emphasizes the necessity for ongoing research and education in this area.

## Introduction

Epistaxis, commonly known as nosebleed, is the most prevalent emergency affecting the ear, nose, and throat (ENT) sector, particularly in pediatric populations [1]. This condition, characterized by bleeding from the nose or nasal cavity, can be broadly categorized into anterior and posterior epistaxis [2]. Anterior epistaxis, often originating from Kiesselbach's plexus, is usually self-limited and more common in children and young adults. In contrast, posterior epistaxis, typically stemming from branches of the sphenopalatine artery in Woodruff's nasopharyngeal plexus, is more prevalent in older adults, especially those with hypertension and arteriosclerosis [2].

Globally, epistaxis impacts around 60% of the population, with approximately 6% seeking medical attention due to the ineffectiveness of traditional home remedies in stopping nasal bleeding [3]. In children under five years, the prevalence is about 30%, increasing to over 50% in children older than five. Notably, in a tertiary teaching hospital in Saudi Arabia, epistaxis-related emergency visits constitute about 1% of all emergency visits [4]. In children, causes of epistaxis range from blood disorders, medications, and trauma to nasopharyngitis, nasal infections or allergies, upper airway infections, and dental inflammation [5,6]. The arid climate of Saudi Arabia, with its low humidity, further exacerbates the incidence of nosebleeds [7]. Epistaxis of a severe or chronic nature can pose significant health risks, potentially leading to life-threatening situations such as shock or even mortality if not managed appropriately. Complications stemming from epistaxis may include anemia, hypovolemia, and shock [8].

The management of patients experiencing active epistaxis necessitates immediate hemostatic intervention and stabilization prior to a comprehensive assessment. The therapeutic approach for stable patients involves a detailed history and examination. Treatment modalities may vary from local to systemic and encompass both surgical and medical strategies, contingent upon the underlying etiology and associated complications [9-12]. Most epistaxis cases occur outside of hospital settings [5], and symptoms can often be managed with basic first aid techniques by non-medical personnel [13]. This study aimed to evaluate the presentation of epistaxis in children in clinical settings, examining the causes and treatment options and determining the associations across demographic variables, causes of epistaxis, and various treatment methods.

## Materials and methods

Study design and setting

This retrospective chart review study was conducted over a period of six months, from March to September 2023, at Al Qurayyat General Hospital in Al Qurayyat, Saudi Arabia. The study protocol was approved by the Research Ethics Committee of the Qurayyat Health Affairs (approval number: 2022-35). Written informed consent was obtained from the patients or their parents before initiating the study.

Participants

Inclusion criteria include all children, both boys and girls, under 16 years of age who presented with epistaxis at the clinics. The study excluded records that are incomplete or lack sufficient details on the treatment of epistaxis.

Variables

Data were extracted from patient medical records. Data were collected on demographic variables, clinical presentation of epistaxis, identified causes, and treatment approaches. Potential biases in the selection of records and reporting of treatment outcomes were addressed by standardizing data extraction protocols and training researchers involved in data collection.

Statistical methods

The sample size, calculated using a chi-squared test with an alpha of 0.05 and a power of 80%, was estimated to be 1451 participants, based on the findings of Reis et al. [14]. Categorical data were presented as frequencies and percentages, while continuous data were presented as mean and standard deviation (SD). Quantitative analysis included demographic characteristics, frequency and duration of epistaxis episodes, and types of treatment administered. The chi-squared test was employed to examine associations between demographic characteristics, epistaxis causes, and treatment approaches. A p-value of <0.05 was considered statistically significant. Data analysis was conducted using IBM SPSS Statistics for Windows, Version 25.0 (Released 2017; IBM Corp., Armonk, New York, United States).

## Results

A total of 219 patients were included in the study; 58% were males and 42% were females. All the included patients (100%) were of Saudi nationality.

Clinical presentations

**Figure 1:**
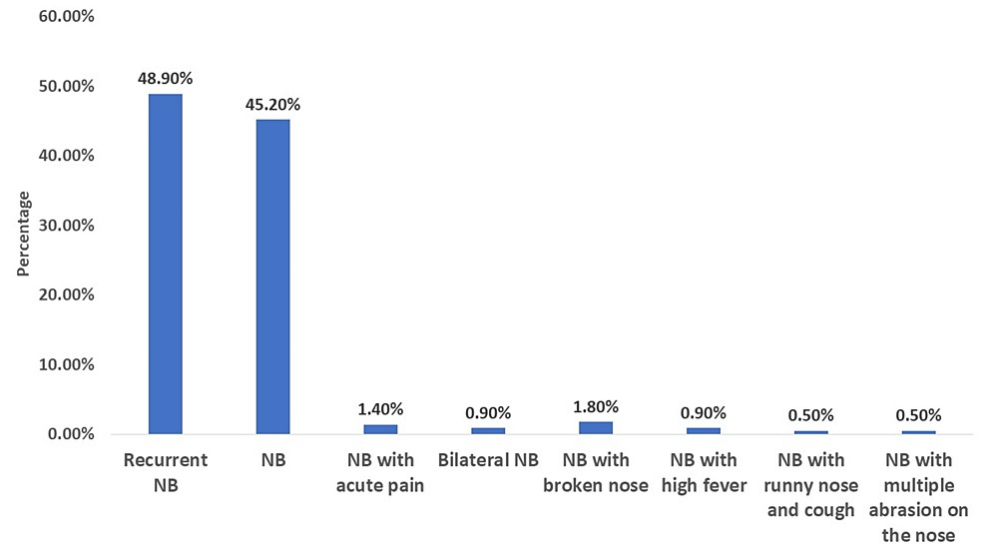
Clinical presentations of included patients NB: nasal bleeding

Recurrent nasal bleeding was the most prevalent, seen in 107 cases (48.9%). This was followed by nasal bleeding without additional symptoms in 99 cases (45.2%). Other presentations were less common: nasal bleeding with acute pain in three cases (1.4%), bilateral nasal bleeding in two cases (0.9%), nasal bleeding with high fever in two cases (0.9%), and nasal bleeding with a broken nose in four cases (1.8%). Rare presentations, each constituting one case (0.5%), included nasal bleeding accompanied by a runny nose and cough, nasal bleeding with multiple abrasions on the nose, and another instance of nasal bleeding with high fever, as shown in Figure 1.

Causes of epistaxis

**Figure 2:**
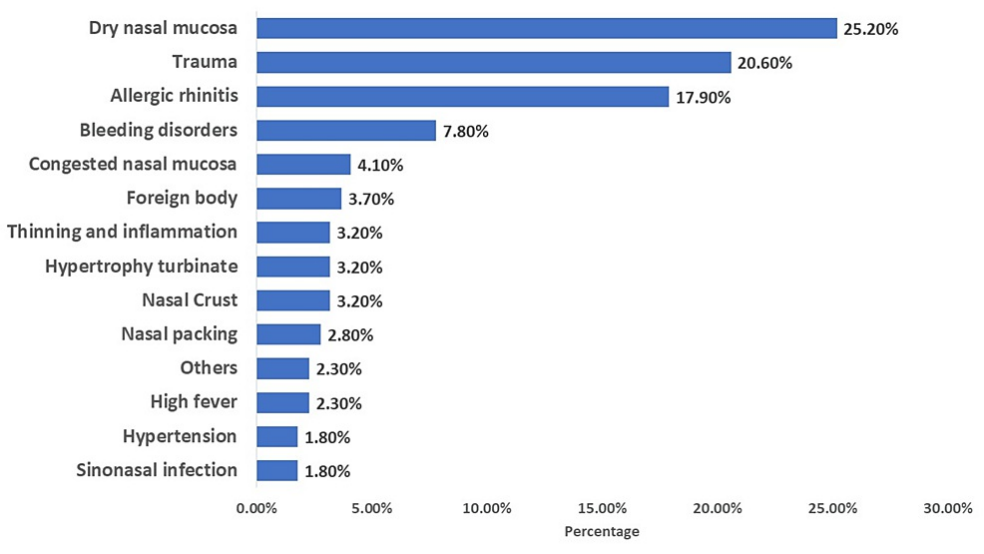
Causes of epistaxis

The most common cause was dry nasal mucosa, observed in 55 cases (25.2%). Trauma was the second most frequent cause, reported in 45 cases (20.6%), followed closely by allergic rhinitis in 39 cases (17.9%). Bleeding disorders were identified in 17 cases (7.8%). Less common causes included congested nasal mucosa (nine cases, 4.1%), foreign body presence (eight cases, 3.7%), nasal crust (seven cases, 3.2%), hypertrophy of the turbinate (seven cases, 3.2%), thinning and inflammation of the nasal mucosa (seven cases, 3.2%), and nasal picking (six cases, 2.8%). Additionally, sinonasal infection and hypertension each were reported in four cases (1.8%), high fever in five cases (2.3%), and other unspecified causes in five cases (2.3%), as shown in Figure 2.

Treatment patterns

**Figure 3:**
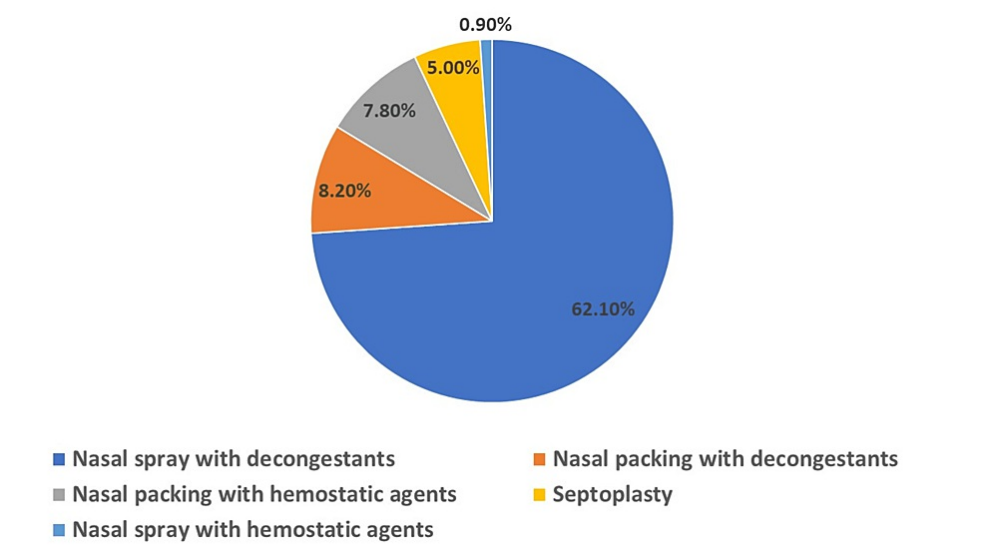
Treatment patterns

Home remedies were reported in 23.74%, while clinical management was reported in 74.88%. First aid management was reported at home in 90% of the epistaxis cases, while 10% applied nasal decompression. In the treatment of epistaxis among pediatric patients, various interventions were employed. The use of nasal sprays containing decongestants was the most common approach, applied in 136 cases (62.1%). Nasal packing with decongestants was also utilized, though less frequently, in 18 cases (8.2%). Similarly, nasal packing with hemostatic agents was used in 17 cases (7.8%). In contrast, nasal sprays containing hemostatic agents were rarely used, reported in only two cases (0.9%). Additionally, septoplasty, a surgical intervention, was performed in 11 cases (5%), as shown in Figure 3.

Comparison between home and clinical settings

In this study comparing the management of epistaxis in home and clinical settings, significant differences were observed in gender distribution, clinical presentation, and treatment approaches. Males comprised 46.2% of the home setting group and 61% of the clinical setting group (p-value=0.06), while females represented 53.8% in the home setting and 39% in the clinical setting.

Regarding clinical presentation, recurrent nasal bleeding was significantly more common in the home setting (75%) compared to the clinical setting (40.2%), with a p-value of <0.001. Other presentations, like isolated nasal bleeding, nasal bleeding with acute pain, bilateral nasal bleeding, nasal bleeding with a broken nose, high fever, runny nose and cough, or multiple abrasions on the nose, showed varying distributions but were generally more prevalent in the clinical setting.

In terms of causes, dry nasal mucosa was more frequently observed in the home setting (34.6%) compared to the clinical setting (21.5%). Trauma and allergic rhinitis were more common in the clinical setting. The other causes, including bleeding disorders, sinonasal infection, high fever, nasal crust, hypertension, foreign body, congested nasal mucosa, hypertrophy of the turbinate, thinning and inflammation, and nasal picking, varied in their distribution but did not show a significant difference.

Treatment methods also differed significantly between settings. The use of nasal spray with decongestants was higher in the clinical setting (70.7%) compared to the home setting (36.5%), with a p-value of <0.001. However, the use of nasal sprays with hemostatic agents, nasal packing with decongestants, and nasal packing with hemostatic agents did not show a significant difference between the two settings (Table 1).

**Table 1:** Comparison between home and clinical settings

Variables		Home setting	Clinical setting	P-value
Gender	Male	24 (46.2%)	100 (61%)	0.06
	Female	28 (53.8%)	64 (39%)	
Clinical presentation	Recurrent nasal bleeding	39 (75%)	66 (40.2%)	<0.001
	Nasal bleeding	11 (21.2%)	87 (53%)	
	Nasal bleeding with acute pain	0 (0%)	3 (1.8%)	
	Bilateral nasal bleeding	1 (1.9%)	1 (0.6%)	
	Nasal bleeding with a broken nose	0 (0%)	4 (2.4%)	
	Nasal bleeding with high fever	0 (0%)	2 (1.2%)	
	Nasal bleeding with runny nose and cough	1 (1.9%)	0 (0%)	
	Nasal bleeding with multiple abrasion on the nose	0 (0%)	1 (0.6%)	
Causes	Bleeding disorders	6 (11.5%)	11 (6.7%)	0.182
	Trauma	6 (11.5%)	38 (23.3%)	
	Allergic rhinitis	10 (19.2%)	29 (17.8%)	
	Sinonasal infection	0 (0%)	4 (2.5%)	
	High fever	0 (0%)	5 (3.1%)	
	Nasal crust	1 (1.9%)	6 (3.7%)	
	Dry nasal mucosa	18 (34.6%)	35 (21.5%)	
	Hypertension	1 (1.9%)	3 (1.8%)	
	Foreign body	1 (1.9%)	7 (4.3%)	
	Congested nasal mucosa	4 (7.7%)	5 (3.1%)	
	Hypertrophy turbinate	2 (3.8%)	5 (3.1%)	
	Thinning and inflammation	3 (5.8%)	4 (2.5%)	
	Nasal packing	0 (0%)	6 (3.7%)	
	Others	0 (0%)	5 (3.1%)	
Nasal spray with decongestants	Yes	19 (36.5%)	116 (70.7%)	<0.001
	No	33 (63.5%)	48 (29.3%)	
Nasal spray with hemostatic agents	Yes	1 (1.9%)	1 (0.6%)	0.389
	No	51 (98.1%)	163 (99.4%)	
Nasal packing with decongestants	Yes	7 (13.5%)	10 (6.1%)	0.086
	No	45 (86.5%)	154 (93.9%)	
Nasal packing with hemostatic agents	Yes	6 (11.5%)	11 (6.7%)	0.26
	No	46 (88.5%)	153 (93.3%)	

## Discussion

In this study, our findings showed that the prevalence of epistaxis in males was higher than in females. Similarly, Passali et al. showed that the prevalence of epistaxis among pediatrics is higher in males compared to females (53.2% vs. 46.8%) [15]. Another study by Sharma et al. demonstrated that the rate of epistaxis in males was 69.08%, while in females, it was 30.92% [16]. This gender distribution aligns with the findings of Hussain et al., who reported in their study of 313 patients that epistaxis is twice as prevalent in males compared to females [17]. Furthermore, the study by Juselius [18] noted a marginally higher incidence of epistaxis in males than in females, which mirrors the trend observed in our research. Such a trend suggests underlying physiological or behavioral factors that may predispose males to a higher risk of epistaxis, warranting further investigation to understand the reasons behind this gender disparity.

Our study highlights a significant prevalence of recurrent nasal bleeding observed in nearly half of the cases (48.9%). This finding highlights the chronic nature of epistaxis in a substantial proportion of the pediatric population, suggesting a need for ongoing medical attention and possibly long-term management strategies. A cross-sectional study involving 1,218 children aged 11-14 years found that approximately 9% of them had frequent episodes of epistaxis, indicating that recurrent epistaxis is common in pediatric populations [19]. Some factors may contribute to recurrent epistaxis in children. For instance, children with migraine headaches may have a higher incidence of recurrent epistaxis compared to those without the disease [20]. The study also sheds light on less frequent clinical presentations of epistaxis. The occurrence of nasal bleeding with acute pain, bilateral nasal bleeding, and nasal bleeding with high fever, although relatively rare (each under 2%), points to a diverse range of clinical manifestations of epistaxis. The rarest presentations, each comprising only 0.5% of cases, included nasal bleeding with runny nose and cough, multiple abrasions on the nose, and high fever. These varied presentations may require differential diagnostic approaches and tailored treatment plans. The diversity in the clinical manifestations of epistaxis highlighted in our study reinforces the importance of individualized patient assessment and emphasizes the need for clinicians to be vigilant for less common but potentially significant presentations of nasal bleeding in pediatric patients [21].

Regarding the causes of epistaxis, we found that the most common causes were dry nasal mucosa, followed by trauma, allergic rhinitis, and bleeding disorders. Dry nasal mucosa is more prone to damage and irritation. When the nasal mucosa becomes dry, it can become fragile and more susceptible to injury, which includes small blood vessels in the nose [22]. Low humidity, especially in dry climates and during cold weather, can contribute to the drying of nasal mucosa. This dry environment can irritate and weaken the mucosa, making it more likely for nosebleeds to occur. Trauma is a recognized risk factor for epistaxis. In pediatric cases, traumatic epistaxis is more prevalent in younger individuals and can be attributed to factors such as digital trauma (nose picking), facial injury, or the presence of a foreign body in the nasal cavity [23]. Allergic rhinitis can lead to recurrent epistaxis, particularly in children [3]. In many cases, nosebleeds are associated with nasal symptoms caused by allergic rhinitis. A study found a significant association between allergic rhinitis and epistaxis in children. They showed that children with positive skin tests had a three times higher risk of epistaxis compared with those with negative tests [24]. Allergies can lead to inflammation and irritation of the nasal passages. This inflammation can make the blood vessels in the nose more susceptible to rupture, resulting in nosebleeds [25]. In terms of bleeding disorders, several studies highlighted their association with epistaxis. Children with recurrent epistaxis, despite medical therapy, are at a higher risk of having a bleeding disorder. A study found that approximately 10.6% of highly selected patients with recurrent epistaxis had underlying bleeding disorders [26]. These risk factors, especially allergic rhinitis and bleeding disorders, should be considered when treating patients with epistaxis to avoid the risk of recurrent epistaxis.

The study reveals a multifaceted approach comprising both home remedies and clinical interventions. Notably, home remedies were reported in 52% of the cases, indicating a significant reliance on initial self-care measures. Within these home treatments, first aid management was predominant, accounting for 90% of the cases, while nasal decompression was utilized in 10%. This trend suggests that while many caregivers are inclined towards immediate, accessible interventions, there is still a portion that opts for more specific techniques like nasal decompression. These findings underscore the importance of educating the public about effective first aid measures for epistaxis to ensure timely and appropriate care [27]. Clinical management of epistaxis showed a higher incidence. Among the clinical interventions, the use of nasal sprays containing decongestants, such as oxymetazoline, was the most common. This preference for decongestant nasal sprays could be attributed to their efficacy in reducing nasal congestion and controlling minor bleeding [28,29]. In contrast, nasal packing with decongestants and hemostatic agents was less frequently used. The utilization of these techniques, although less common, highlights their role in more severe or persistent cases of epistaxis where simple decongestant sprays may not be sufficient.

Recurrent nasal bleeding was significantly more common in the home setting than in the clinical setting, indicating that recurrent cases might be more frequently managed at home, possibly due to familiarity with the condition or its perceived lesser severity. Regarding treatment methods, the use of nasal spray with decongestants was substantially higher in the clinical setting than in the home setting, highlighting a preference for professional medical management in more severe or persistent cases. Conversely, the use of nasal sprays with hemostatic agents and nasal packing with decongestants or hemostatic agents did not show significant differences between settings, suggesting these treatments are less influenced by the setting and more by the specific clinical requirements of each epistaxis case.

Limitations

We acknowledge that the study has several limitations. Firstly, the retrospective design of the study inherently limits the ability to establish causality between observed factors and epistaxis outcomes. Secondly, the reliance on existing medical records for data extraction may have introduced selection bias, as cases with incomplete or less detailed records were possibly overlooked, potentially skewing the findings. Another limitation is the potential for reporting bias, especially in the context of home treatment methods. The accuracy and completeness of self-reported home management practices could vary, affecting the reliability of data regarding home treatments. Additionally, the study's sample size and geographic scope may limit the generalizability of the findings. Furthermore, the study did not account for all possible confounding factors, such as socioeconomic status, access to healthcare, and cultural practices, which could significantly influence the management of epistaxis. These factors might affect the choice of home versus clinical management and the types of interventions used in different settings. Lastly, the study did not differentiate between the severity of epistaxis episodes, which could be a crucial factor in determining the choice of a treatment strategy. Future studies could benefit from a more comprehensive approach, including a prospective design, broader demographic representation, and consideration of confounding factors to provide a more nuanced understanding of pediatric epistaxis management.

## Conclusions

The study provides valuable insights into the management of pediatric epistaxis, highlighting notable differences in approaches between home and clinical settings. It was observed that home remedies are commonly employed, with a significant reliance on first aid measures. In clinical settings, the use of nasal sprays containing decongestants was predominant, indicating a preference for medical interventions in more severe cases. The gender distribution differed between settings, with a higher proportion of males in clinical settings, suggesting potential gender-based differences in the occurrence or management of epistaxis. Furthermore, the study emphasizes the diverse nature of epistaxis presentations and the range of treatment methods employed. While recurrent nasal bleeding was more common in home settings, more complex cases such as nasal bleeding with acute pain or broken nose were more likely to be treated in clinical environments. This indicates the importance of tailored management strategies based on the severity and frequency of epistaxis episodes. There is a need for continued research and education in the effective management of pediatric epistaxis. Healthcare providers should be aware of the various treatment options and consider individual patient circumstances, especially when deciding between home-based and clinical interventions.

## References

[1] Adoga AA, Kokong DD, Mugu JG, Okwori ET, Yaro JP (2019). Epistaxis: the demographics, etiology, management, and predictors of outcome in Jos, North-Central Nigeria. Ann Afr Med.

[2] Dubel GJ, Ahn SH, Soares GM (2013). Transcatheter embolization in the management of epistaxis. Semin Intervent Radiol.

[3] Shay S, Shapiro NL, Bhattacharyya N (2017). Epidemiological characteristics of pediatric epistaxis presenting to the emergency department. Int J Pediatr Otorhinolaryngol.

[4] Alharethy SE (2019). Recent insight into the prevalence, etiology, and outcome of epistaxis in a university hospital in Saudi Arabia. J Nat Sci Med.

[5] Alasiri AS, Magboul NA, Alasiri AB, Al-Amri D, Albarqi HH, AlAlhareth MS, Alshandari T (2022). Teacher's awareness regarding epistaxis first-aid management inside schools in Asser Region, Saudi Arabia. Egypt J Otolaryngol.

[6] Alqarni ZM, Alajmi TA, Alhumaidi HH, Alhussain A, Alotaibi YM, Alzahrani HS (2019). Prevalence, causes, treatment, and outcome of epistaxis. Int J Med Dev Ctries.

[7] Alhaddad MS, Almulhim K, Mubarak IA, Alotaibi N, Hussain MA, Alyahya KA (2017). Prevalence of epistaxis in Saudi population. Int J Sci Study.

[8] Adegbiji WA, Olajide GT, Olatoke F, Nwawolo CC (2018). Clinico-epidemiological pattern and treatment of epistaxis in a tertiary hospital in South Western Nigeria. Int J Otorhinolaryngol Head Neck Surg.

[9] Bernius M, Perlin D (2006). Pediatric ear, nose, and throat emergencies. Pediatr Clin North Am.

[10] Middleton PM (2004). Epistaxis. Emerg Med Australas.

[11] Daudia A, Jaiswal V, Jones NS (2008). Guidelines for the management of idiopathic epistaxis in adults: how we do it. Clin Otolaryngol.

[12] Gilyoma JM, Chalya PL (2011). Etiological profile and treatment outcome of epistaxis at a tertiary care hospital in Northwestern Tanzania: a prospective review of 104 cases. BMC Ear Nose Throat Disord.

[13] Mohammad SM, Alsharidah AA, Alshehri M (2020). Knowledge and practice of epistaxis first aid among adult population in Riyadh, Saudi Arabia. Int J Med Dev Ctries.

[14] Reis LR, Correia F, Castelhano L, Escada P (2018). Epidemiology of epistaxis in the emergency department of a southern European tertiary care hospital. Acta Otorrinolaringol Esp (Engl Ed).

[15] Passali D, Damiani V, Passali FM, Tosca MA, Motta G, Ciprandi G (2020). An international survey on the pragmatic management of epistaxis. Acta Biomed.

[16] Sharma S, Qureshi S, Jadia S, Ukawat L (2020). Epistaxis: revisited. Indian J Otolaryngol Head Neck Surg.

[17] Hussain G, Iqbal M, Shah SA (2006). Evaluation of aetiology and efficacy of management protocol of epistaxis. J Ayub Med Coll Abbottabad.

[18] Juselius H (1974). Epistaxis. A clinical study of 1,724 patients. J Laryngol Otol.

[19] McGarry GW (2013). Recurrent epistaxis in children. BMJ Clin Evid.

[20] Tunkel DE, Anne S, Payne SC (2020). Clinical practice guideline: nosebleed (epistaxis) executive summary. Otolaryngol Head Neck Surg.

[21] Senthilkumar AT, Jeba GA (2021). Epistaxis nose-bleed. Int J Hom Sci.

[22] Selvarajah J, Saim AB, Bt Hj Idrus R, Lokanathan Y (2020). Current and alternative therapies for nasal mucosa injury: a review. Int J Mol Sci.

[23] Fatakia A, Winters R, Amedee RG (2010). Epistaxis: a common problem. Ochsner J.

[24] Murray AB, Milner RA (1995). Allergic rhinitis and recurrent epistaxis in children. Ann Allergy Asthma Immunol.

[25] Teo WY, Wong HB, Hwarng GY, Tan HK (2023). Outcome of childhood epistaxis with treatment of allergic rhinitis: a randomized controlled study. Eur J Pediatr.

[26] Elden L, Reinders M, Witmer C (2012). Predictors of bleeding disorders in children with epistaxis: value of preoperative tests and clinical screening. Int J Pediatr Otorhinolaryngol.

[27] Abd El-Alim Ebrahim N, Abd El-Aziz Abd El-Salam A, Abd ElGhany Mohamed R, El-Sayed Metwally H (2022). Effect of educational guidelines on mothers' performance regarding care of children with epistaxis. J Nurs Sci Benha Univ.

[28] Smith J, Hanson J, Chowdhury R, Bungard TJ (2019). Community-based management of epistaxis: who bloody knows?. Can Pharm J (Ott).

[29] Koçak HE, Bilece ZT, Keskin M, Ulusoy HA, Koç AK, Kaya KH (2021). Comparison of topical treatment methods used in recurrent anterior epistaxis: a randomized clinical trial. Braz J Otorhinolaryngol.

